# Crystal structure of *N* ^1^-benzyl-*N* ^1^,*N* ^2^,*N* ^2^-tri­methyl­ethane-1,2-diaminium dichloride

**DOI:** 10.1107/S1600536814015797

**Published:** 2014-08-01

**Authors:** Pushpendra Singh, Harkesh B. Singh, Ray J. Butcher

**Affiliations:** a Department of Chemistry, Indian Institute of Technology Bombay, Powai, Mumbai, 400076, India; b Department of Chemistry, Howard University, 525 College Street NW, Washington DC 20059, USA

**Keywords:** crystal structure, hydrogen bonds, C—H⋯Cl inter­actions, ion-triplets, inversion twin

## Abstract

In the title mol­ecular salt, C_12_H_22_N_2_^2+^·2Cl^−^, which was obtained as a by-product in the attempted synthesis of a mercury derivative, the conformation of the N—C—C—N bond in the cation is *anti* [torsion angle = 175.1 (10)°]. In the crystal, the cations are linked to the anions by N—H⋯Cl hydrogen bonds, generating ion-triplets. These are linked by numerous weak C—H⋯Cl inter­actions, generating a three-dimensional network. The structure was refined as an inversion twin.

## Related literature   

For further synthetic details, see: Rietveld *et al.* (1994 ▶). For the application of the parent di­amine as a precursor of anti-histamine derivatives for therapeutic use, see: Gardner & Stevens (1949 ▶); Fox & Wenner (1951 ▶); For a related structure, see: Manjare *et al.* (2014 ▶).
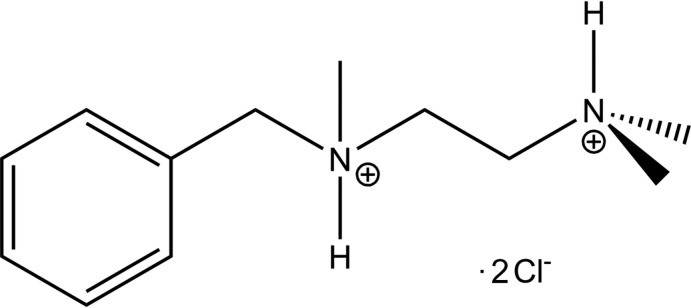


## Experimental   

### Crystal data  

C_12_H_22_N_2_^2+^·2Cl^−^
*M* *_r_* = 265.21
Monoclinic, 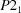

*a* = 5.6744 (7) Å
*b* = 22.384 (3) Å
*c* = 5.9991 (7) Å
β = 105.372 (12)°
*V* = 734.72 (16) Å^3^
*Z* = 2
Cu *K*α radiation
μ = 3.79 mm^−1^
*T* = 123 K
0.49 × 0.16 × 0.13 mm

### Data collection  

Agilent Xcalibur, Ruby, Gemini diffractometer
Absorption correction: multi-scan (*CrysAlis PRO*; Agilent, 2012 ▶) *T*_min_ = 0.273, *T*_max_ = 1.000
2002 measured reflections
1986 independent reflections
1961 reflections with *I* > 2σ(*I*)
*R*_int_ = 0.000

### Refinement  

*R*[*F* ^2^ > 2σ(*F* ^2^)] = 0.082
*wR*(*F* ^2^) = 0.229
*S* = 1.13
1986 reflections
149 parameters
7 restraints
H-atom parameters constrained
Δρ_max_ = 1.15 e Å^−3^
Δρ_min_ = −0.62 e Å^−3^
Refined as an inversion twin
Absolute structure parameter: 0.25 (7)

### 

Data collection: *CrysAlis PRO* (Agilent, 2012 ▶); cell refinement: *CrysAlis PRO* (Agilent, 2012 ▶); data reduction: *CrysAlis PRO* (Agilent, 2012 ▶); program(s) used to solve structure: *SHELXS97* (Sheldrick, 2008 ▶); program(s) used to refine structure: *SHELXL2013* (Sheldrick, 2008 ▶); molecular graphics: *SHELXTL* (Sheldrick, 2008 ▶); software used to prepare material for publication: *SHELXTL* (Sheldrick, 2008 ▶).

## Supplementary Material

Crystal structure: contains datablock(s) I. DOI: 10.1107/S1600536814015797/hb7244sup1.cif

Structure factors: contains datablock(s) I. DOI: 10.1107/S1600536814015797/hb7244Isup2.hkl

Supporting information file. DOI: 10.1107/S1600536814015797/hb7244Isup3.cml

**Fig. 1.**
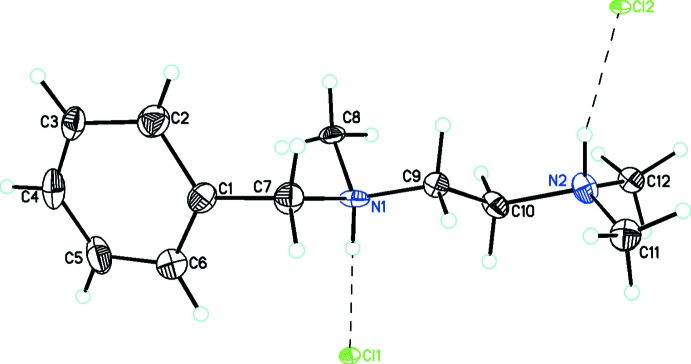
The molecular structure of C12H22N2.2Cl showing 30% probability displacement ellipsoids and the N—H···Cl hydrogen bonds (shown as dashed lines).

12 22 2 . DOI: 10.1107/S1600536814015797/hb7244fig1.tif
The mol­ecular structure of C_12_H_22_N_2_·2Cl showing 30% probability displacement ellipsoids and the N—H⋯Cl hydrogen bonds (shown as dashed lines).

**Fig. 2.**
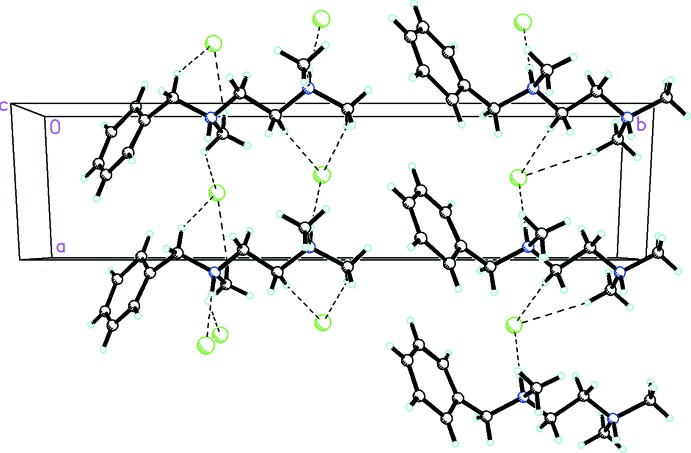
The packing for C12H22N2.2Cl viewed along the b axis showing the linking of the cations and anions into a three-dimensional array by an extensive network of C—H···Cl interactions (shown as dashed bonds).

12 22 2 b . DOI: 10.1107/S1600536814015797/hb7244fig2.tif
The packing for C_12_H_22_N_2_·2Cl viewed along the *b* axis showing the linking of the cations and anions into a three-dimensional array by an extensive network of C—H⋯Cl inter­actions (shown as dashed bonds).

CCDC reference: 1012363

Additional supporting information:  crystallographic information; 3D view; checkCIF report

## Figures and Tables

**Table 1:** Hydrogen-bond geometry (Å, °)

*D*—H⋯*A*	*D*—H	H⋯*A*	*D*⋯*A*	*D*—H⋯*A*
N1—H1*A*⋯Cl1	1.00	2.11	3.107 (8)	179
N2—H2*B*⋯Cl2	1.00	2.18	3.148 (11)	163
C7—H7*B*⋯Cl1^i^	0.99	2.92	3.749 (13)	142
C8—H8*A*⋯Cl2^ii^	0.98	2.93	3.893 (10)	168
C8—H8*B*⋯Cl1^i^	0.98	2.79	3.690 (11)	154
C8—H8*C*⋯Cl1^iii^	0.98	2.88	3.486 (12)	121
C9—H9*A*⋯Cl1^iv^	0.99	2.74	3.711 (12)	168
C10—H10*A*⋯Cl1	0.99	2.98	3.682 (11)	129
C10—H10*B*⋯Cl2^ii^	0.99	2.78	3.750 (13)	168
C11—H11*B*⋯Cl1^iv^	0.98	2.89	3.831 (17)	161
C12—H12*B*⋯Cl2^ii^	0.98	2.89	3.842 (15)	166
C12—H12*C*⋯Cl2^v^	0.98	2.88	3.747 (13)	147

Symmetry codes: (i) 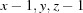; (ii) 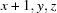; (iii) 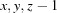; (iv) 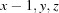; (v) 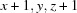.

## supplementary crystallographic information

### S2. Experimental

The starting material, *o*-di­amine-substituted aryl bromide, *N^1^*-(2-bromo­benzyl)-*N^1^*,*N^2^*,*N^2^*-tri­methyl­ethane-1,2-di­amine, can be prepared by the reaction of *N*^1^,*N*^1^,*N*^2^-tri­methyl­ethane-1,2-di­amine and *ortho*-bromo­benzyl­bromide (Rietveld *et al.*, 1994). This ligand is moisture sensitive and is difficult to purify by column chromatography. However, it could be easily purified by vacuum distillation. The moisture sensitive ligand when treated with *n*-BuLi in THF produced the li­thia­ted product (2) which when treated with AlCl_3_ afforded the title salt.

A stirred solution of *N^1^*-(2-bromo­benzyl)-*N^1^*,*N^2^*,*N^2^*-tri­methyl­ethane-1,2-di­amine (1.10 ml, 5.34 mmol) in dry THF (15 ml) was treated dropwise with a 1.6 M solution of *n*-BuLi in hexane (3.80 ml, 6.15 mmol) via syringe under N_2_ at 0°C. On stirring the reaction mixture for 2 h at this temperature the li­thia­ted product (2) was obtained. To a freshly prepared 2 (1.10 ml, 5.34 mmol) in dry THF (15 ml) was added anhydrous aluminum trichloride (0.75 g, 5.70 mmol) under a brisk flow of N_2_ gas and stirring was continued for an additional 6 h at room temperature. The reaction mixture was then removed from the N_2_ line and evaporated to dryness to give a colourless hygroscopic solid. The solid was extracted with dry ether. The organic phase was separated, dried over Na_2_SO_4_, and filtered. The filtrate was evaporated to dryness to give a colourless crystalline solid of the title salt (0.48, 34% yield). ^1^H NMR (CDCl_3_) *δ* 2.72 (s, NMe_2_), 2.38 (s, NMe), 2.95 (t, 2H), 3.13 (3, 2H), 3.65 (s, CH_2_), 5.48 (s, br 2NH), 7.31-7.39 (m, 5H-aryl); ^13^C NMR (DMSO-d_6_) *δ* 41.52, 43.40, 51.86, 54.32, 61.69, 67. 34, 127.50, 128.51, 129.43, 138.18.

### S2.1. Refinement

H atoms were placed in geometrically idealized positions and constrained to ride on their parent atoms with a C—H distances of 0.95 and 0.99 Å and an N—H distance of 1.00 *U*_iso_(H) = 1.2*U*_eq_(C, N) and 0.98 Å for CH_3_ [*U*_iso_(H)= 1.5*U*_eq_(C)]

### Crystal data

**Table:**

C_12_H_22_N_2_^2+^·2Cl^−^	*F*(000) = 284
*M**_r_* = 265.21	*D*_x_ = 1.199 Mg m^−^^3^
Monoclinic, *P*2_1_	Cu *K*α radiation, λ = 1.54178 Å
*a* = 5.6744 (7) Å	Cell parameters from 1555 reflections
*b* = 22.384 (3) Å	θ = 4.0–76.4°
*c* = 5.9991 (7) Å	µ = 3.79 mm^−^^1^
β = 105.372 (12)°	*T* = 123 K
*V* = 734.72 (16) Å^3^	Needle, colorless
*Z* = 2	0.49 × 0.16 × 0.13 mm

### Data collection

**Table:**

Agilent Xcalibur, Ruby, Gemini diffractometer	1961 reflections with *I* > 2σ(*I*)
Detector resolution: 10.5081 pixels mm^-1^	*R*_int_ = 0.000
ω scans	θ_max_ = 76.6°, θ_min_ = 4.0°
Absorption correction: multi-scan (*CrysAlis PRO*; Agilent, 2012)	*h* = −7→6
*T*_min_ = 0.273, *T*_max_ = 1.000	*k* = −27→21
2002 measured reflections	*l* = 0→7
1986 independent reflections	

### Refinement

**Table:**

Refinement on *F*^2^	Secondary atom site location: difference Fourier map
Least-squares matrix: full	Hydrogen site location: inferred from neighbouring sites
*R*[*F*^2^ > 2σ(*F*^2^)] = 0.082	H-atom parameters constrained
*wR*(*F*^2^) = 0.229	*w* = 1/[σ^2^(*F*_o_^2^) + (0.1591*P*)^2^ + 1.0863*P*] where *P* = (*F*_o_^2^ + 2*F*_c_^2^)/3
*S* = 1.13	(Δ/σ)_max_ < 0.001
1986 reflections	Δρ_max_ = 1.15 e Å^−^^3^
149 parameters	Δρ_min_ = −0.62 e Å^−^^3^
7 restraints	Absolute structure: Refined as an inversion twin.
Primary atom site location: structure-invariant direct methods	Absolute structure parameter: 0.25 (7)

### Special details

**Table:**

Geometry. All e.s.d.'s (except the e.s.d. in the dihedral angle between two l.s. planes) are estimated using the full covariance matrix. The cell e.s.d.'s are taken into account individually in the estimation of e.s.d.'s in distances, angles and torsion angles; correlations between e.s.d.'s in cell parameters are only used when they are defined by crystal symmetry. An approximate (isotropic) treatment of cell e.s.d.'s is used for estimating e.s.d.'s involving l.s. planes.
Refinement. Refined as a 2-component inversion twin.

### Fractional atomic coordinates and isotropic or equivalent isotropic displacement parameters (Å^2^)

**Table:**

	*x*	*y*	*z*	*U*_iso_*/*U*_eq_	
Cl1	0.5443 (3)	0.30227 (7)	1.3350 (2)	0.0313 (4)	
Cl2	−0.5635 (2)	0.48206 (5)	0.67629 (19)	0.0219 (3)	
N1	0.0818 (16)	0.3087 (4)	0.9195 (14)	0.052 (2)	
H1A	0.2296	0.3060	1.0540	0.063*	
N2	−0.0884 (18)	0.4633 (5)	1.083 (2)	0.061 (2)	
H2B	−0.2225	0.4640	0.9363	0.074*	
C1	0.092 (2)	0.1998 (6)	0.814 (2)	0.065 (3)	
C2	−0.012 (3)	0.1787 (6)	0.583 (2)	0.078 (4)	
H2A	−0.1656	0.1919	0.4903	0.094*	
C3	0.141 (3)	0.1344 (6)	0.500 (2)	0.075 (3)	
H3A	0.0818	0.1180	0.3493	0.090*	
C4	0.359 (3)	0.1172 (6)	0.629 (3)	0.092 (4)	
H4A	0.4563	0.0907	0.5670	0.110*	
C5	0.446 (3)	0.1374 (7)	0.855 (3)	0.094 (5)	
H5A	0.6003	0.1239	0.9458	0.113*	
C6	0.310 (2)	0.1772 (6)	0.950 (2)	0.074 (3)	
H6A	0.3662	0.1887	1.1075	0.088*	
C7	−0.044 (3)	0.2487 (6)	0.896 (2)	0.067 (3)	
H7A	−0.0677	0.2372	1.0479	0.080*	
H7B	−0.2077	0.2525	0.7861	0.080*	
C8	0.171 (2)	0.3228 (4)	0.7060 (17)	0.050 (2)	
H8A	0.2488	0.3622	0.7239	0.074*	
H8B	0.0318	0.3227	0.5683	0.074*	
H8C	0.2896	0.2925	0.6890	0.074*	
C9	−0.065 (2)	0.3554 (5)	0.967 (2)	0.057 (3)	
H9A	−0.1454	0.3416	1.0862	0.069*	
H9B	−0.1955	0.3646	0.8253	0.069*	
C10	0.079 (2)	0.4127 (6)	1.0534 (19)	0.058 (3)	
H10A	0.1997	0.4047	1.2031	0.069*	
H10B	0.1706	0.4249	0.9411	0.069*	
C11	−0.205 (3)	0.4576 (7)	1.257 (3)	0.075 (4)	
H11A	−0.0833	0.4579	1.4077	0.113*	
H11B	−0.2958	0.4198	1.2383	0.113*	
H11C	−0.3186	0.4910	1.2494	0.113*	
C12	0.045 (3)	0.5223 (6)	1.088 (2)	0.066 (3)	
H12A	−0.0741	0.5546	1.0381	0.099*	
H12B	0.1517	0.5202	0.9838	0.099*	
H12C	0.1441	0.5304	1.2459	0.099*	

### Atomic displacement parameters (Å^2^)

**Table:**

	*U*^11^	*U*^22^	*U*^33^	*U*^12^	*U*^13^	*U*^23^
Cl1	0.0262 (7)	0.0399 (11)	0.0259 (7)	−0.0014 (7)	0.0034 (6)	0.0010 (6)
Cl2	0.0176 (6)	0.0285 (7)	0.0176 (6)	−0.0026 (6)	0.0012 (4)	0.0048 (5)
N1	0.049 (4)	0.063 (5)	0.038 (4)	−0.010 (4)	−0.001 (3)	0.007 (4)
N2	0.044 (4)	0.060 (6)	0.084 (6)	0.002 (4)	0.024 (4)	0.005 (4)
C1	0.065 (6)	0.067 (7)	0.067 (6)	0.001 (5)	0.026 (5)	−0.006 (5)
C2	0.082 (9)	0.080 (8)	0.065 (7)	−0.004 (7)	0.007 (6)	−0.004 (6)
C3	0.091 (8)	0.053 (6)	0.081 (7)	−0.008 (7)	0.023 (6)	−0.012 (6)
C4	0.107 (10)	0.045 (6)	0.143 (13)	0.000 (7)	0.067 (9)	−0.017 (7)
C5	0.084 (9)	0.055 (6)	0.130 (12)	0.005 (8)	0.006 (8)	−0.012 (9)
C6	0.067 (7)	0.083 (9)	0.071 (7)	−0.003 (6)	0.019 (6)	0.002 (6)
C7	0.076 (7)	0.070 (7)	0.060 (6)	−0.003 (6)	0.025 (5)	−0.002 (5)
C8	0.060 (6)	0.049 (6)	0.034 (4)	−0.005 (4)	0.002 (4)	0.002 (3)
C9	0.050 (5)	0.063 (6)	0.060 (6)	0.006 (5)	0.016 (5)	0.003 (5)
C10	0.050 (5)	0.065 (6)	0.049 (5)	0.007 (5)	−0.004 (4)	0.009 (4)
C11	0.056 (6)	0.086 (8)	0.078 (7)	−0.009 (6)	0.007 (6)	−0.018 (6)
C12	0.061 (6)	0.068 (7)	0.063 (7)	0.011 (6)	0.006 (5)	0.006 (5)

### Geometric parameters (Å, º)

**Table:**

N1—C9	1.413 (15)	C5—H5A	0.9500
N1—C7	1.511 (17)	C6—H6A	0.9500
N1—C8	1.530 (14)	C7—H7A	0.9900
N1—H1A	1.0000	C7—H7B	0.9900
N2—C11	1.38 (2)	C8—H8A	0.9800
N2—C10	1.520 (16)	C8—H8B	0.9800
N2—C12	1.521 (17)	C8—H8C	0.9800
N2—H2B	1.0000	C9—C10	1.538 (16)
C1—C6	1.382 (18)	C9—H9A	0.9900
C1—C2	1.435 (18)	C9—H9B	0.9900
C1—C7	1.497 (18)	C10—H10A	0.9900
C2—C3	1.49 (2)	C10—H10B	0.9900
C2—H2A	0.9500	C11—H11A	0.9800
C3—C4	1.33 (2)	C11—H11B	0.9800
C3—H3A	0.9500	C11—H11C	0.9800
C4—C5	1.39 (2)	C12—H12A	0.9800
C4—H4A	0.9500	C12—H12B	0.9800
C5—C6	1.40 (2)	C12—H12C	0.9800
			
C9—N1—C7	112.7 (9)	C1—C7—H7B	108.7
C9—N1—C8	111.3 (9)	N1—C7—H7B	108.7
C7—N1—C8	111.0 (8)	H7A—C7—H7B	107.6
C9—N1—H1A	107.2	N1—C8—H8A	109.5
C7—N1—H1A	107.2	N1—C8—H8B	109.5
C8—N1—H1A	107.2	H8A—C8—H8B	109.5
C11—N2—C10	117.4 (11)	N1—C8—H8C	109.5
C11—N2—C12	113.6 (11)	H8A—C8—H8C	109.5
C10—N2—C12	109.0 (9)	H8B—C8—H8C	109.5
C11—N2—H2B	105.2	N1—C9—C10	113.1 (9)
C10—N2—H2B	105.2	N1—C9—H9A	109.0
C12—N2—H2B	105.2	C10—C9—H9A	109.0
C6—C1—C2	121.6 (13)	N1—C9—H9B	109.0
C6—C1—C7	122.1 (12)	C10—C9—H9B	109.0
C2—C1—C7	116.3 (12)	H9A—C9—H9B	107.8
C1—C2—C3	114.6 (13)	N2—C10—C9	111.4 (9)
C1—C2—H2A	122.7	N2—C10—H10A	109.3
C3—C2—H2A	122.7	C9—C10—H10A	109.3
C4—C3—C2	122.1 (13)	N2—C10—H10B	109.3
C4—C3—H3A	118.9	C9—C10—H10B	109.3
C2—C3—H3A	118.9	H10A—C10—H10B	108.0
C3—C4—C5	120.6 (15)	N2—C11—H11A	109.5
C3—C4—H4A	119.7	N2—C11—H11B	109.5
C5—C4—H4A	119.7	H11A—C11—H11B	109.5
C4—C5—C6	121.0 (14)	N2—C11—H11C	109.5
C4—C5—H5A	119.5	H11A—C11—H11C	109.5
C6—C5—H5A	119.5	H11B—C11—H11C	109.5
C1—C6—C5	119.7 (13)	N2—C12—H12A	109.5
C1—C6—H6A	120.1	N2—C12—H12B	109.5
C5—C6—H6A	120.1	H12A—C12—H12B	109.5
C1—C7—N1	114.2 (11)	N2—C12—H12C	109.5
C1—C7—H7A	108.7	H12A—C12—H12C	109.5
N1—C7—H7A	108.7	H12B—C12—H12C	109.5
			
C6—C1—C2—C3	−4 (2)	C2—C1—C7—N1	−108.9 (14)
C7—C1—C2—C3	174.3 (12)	C9—N1—C7—C1	172.2 (10)
C1—C2—C3—C4	−1 (2)	C8—N1—C7—C1	46.6 (13)
C2—C3—C4—C5	4 (2)	C7—N1—C9—C10	165.1 (9)
C3—C4—C5—C6	−1 (3)	C8—N1—C9—C10	−69.5 (11)
C2—C1—C6—C5	7 (2)	C11—N2—C10—C9	67.8 (13)
C7—C1—C6—C5	−171.5 (14)	C12—N2—C10—C9	−161.2 (10)
C4—C5—C6—C1	−4 (2)	N1—C9—C10—N2	175.1 (10)
C6—C1—C7—N1	69.3 (16)		

### Hydrogen-bond geometry (Å, º)

**Table:**

*D*—H···*A*	*D*—H	H···*A*	*D*···*A*	*D*—H···*A*
N1—H1*A*···Cl1	1.00	2.11	3.107 (8)	179
N2—H2*B*···Cl2	1.00	2.18	3.148 (11)	163
C7—H7*B*···Cl1^i^	0.99	2.92	3.749 (13)	142
C8—H8*A*···Cl2^ii^	0.98	2.93	3.893 (10)	168
C8—H8*B*···Cl1^i^	0.98	2.79	3.690 (11)	154
C8—H8*C*···Cl1^iii^	0.98	2.88	3.486 (12)	121
C9—H9*A*···Cl1^iv^	0.99	2.74	3.711 (12)	168
C10—H10*A*···Cl1	0.99	2.98	3.682 (11)	129
C10—H10*B*···Cl2^ii^	0.99	2.78	3.750 (13)	168
C11—H11*B*···Cl1^iv^	0.98	2.89	3.831 (17)	161
C12—H12*B*···Cl2^ii^	0.98	2.89	3.842 (15)	166
C12—H12*C*···Cl2^v^	0.98	2.88	3.747 (13)	147

Symmetry codes: (i) *x*−1, *y*, *z*−1; (ii) *x*+1, *y*, *z*; (iii) *x*, *y*, *z*−1; (iv) *x*−1, *y*, *z*; (v) *x*+1, *y*, *z*+1.
