# Supplementary figures and images for: Crystal structure of N 1-benzyl-N 1,N 2,N 2-tri­methyl­ethane-1,2-diaminium dichloride
Source: Acta Crystallogr Sect E Struct Rep Online. 2014 Aug 1;70(Pt 9):o911–2. doi: 10.1107/S1600536814015797 (PMC4186135; doi:10.1107/S1600536814015797)

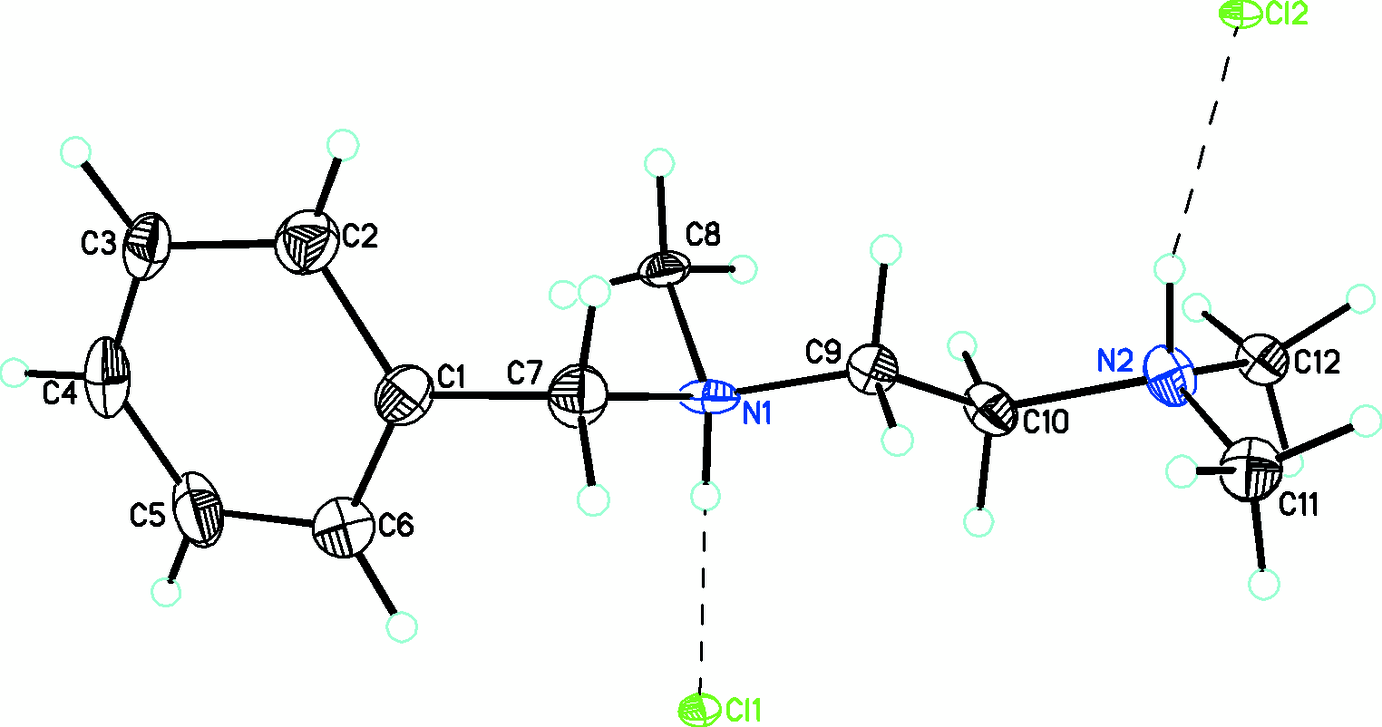

Supplement: Supplementary file 4 [file e-70-0o911-fig1.tif]

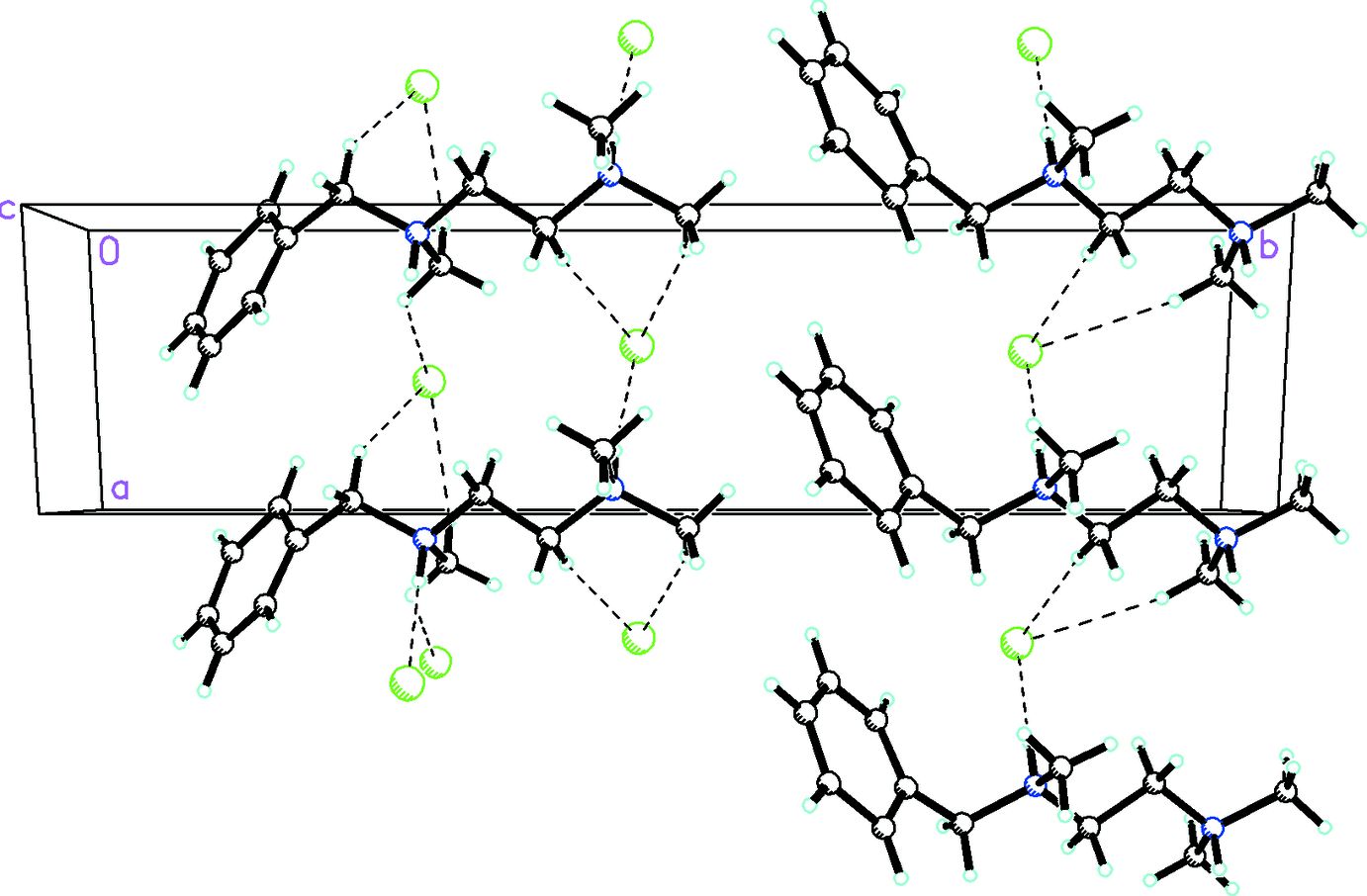

Supplement: Supplementary file 5 [file e-70-0o911-fig2.tif]
